# Community education and health promotion activities of naturopathic practitioners: results of an international cross-sectional survey

**DOI:** 10.1186/s12906-021-03467-z

**Published:** 2021-11-30

**Authors:** Amie Steel, Iva Lloyd

**Affiliations:** 1 Australian Research Centre in Complementary and Integrative Medicine, Faculty of Health, University of Technology Sydney, 235-253 Jones St, Ultimo, Sydney, NSW 2006 Australia; 2 World Naturopathic Federation, Toronto, Canada

**Keywords:** Naturopathy, Health promotion, Patient education, Health education, Survey, Complementary therapies

## Abstract

**Background:**

Health promotion and patient education are crucial to improved population health and are also among the core principles that define naturopathy. Yet, the activities of naturopathic practitioners (NPs) with regards to health promotion and community education have not been widely studied.

**Methods:**

A cross-sectional online survey of an international convenience sample of NPs was conducted through disseminating a 15-item questionnaire prepared in five languages. Correlates of most frequently mentioned NP activities were studied.

**Results:**

The survey was completed by 813 NPs representing all world regions. Almost all participants (98%) reported at least one health promotion activity. Most reported were information sheets and handouts (92.7%) or social and professional network communications (91.8%) and information talks presented to community members (84.9%). The majority of NPs (79.5%) indicated that the ‘health issues individuals in NPs’ community have said they need help with’ were a ‘very important’ consideration when they designed health promotion activities. NP characteristics associated with the likelihood of engaging in specific health promotion activities varied between activities but include gender, time since first qualification, factors considered to identify need when designing an activity, and stakeholder involvement in activity design.

**Conclusions:**

Health promotion is a key activity of the global naturopathic profession. There are a wide range of patient education tools utilized by NPs.

**Supplementary Information:**

The online version contains supplementary material available at 10.1186/s12906-021-03467-z.

## Background

The success of primary health care strategies and health outcomes lies with one’s ability to engage and empower individuals and communities [1]. Health literacy is an identified, and critical, predictor of empowerment, and health education is a tool often employed to improve the health literacy of a population [2]. Health education is a complex process that encompasses the acquisition of health knowledge, the skills required to make informed health decisions, and the motivation to foster positive health behaviours [3]. For this reason, health education activities must be customized to the target population to ensure accessibility and relevance, while also supported by programs that enable individuals from low socioeconomic or marginalized backgrounds to modify health behaviours as needed [4, 5]. Health education can be delivered through diverse channels including mass media, social media, group discussions or presentations, and printed materials [6, 7]. While various social media platforms may be used by patients and health professionals to communicate health information [8, 9], patients also value information received directly from their health care provider [10]. Current evidence indicates positive change in a community’s health behaviours is best achieved through the use of multiple communication activities and channels that direct specific messages to target populations [11, 12].

**Fig. 1:**
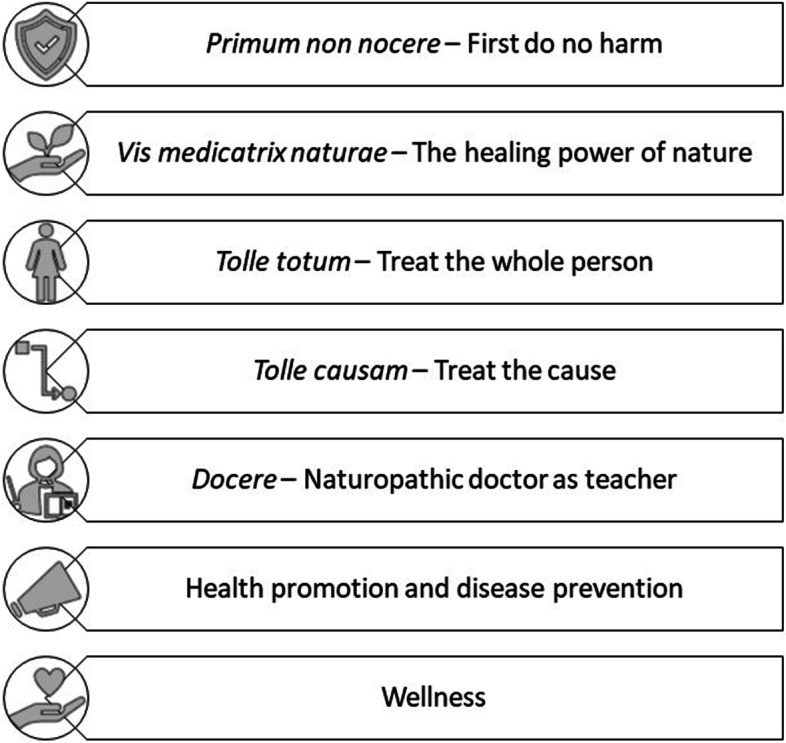
The guiding principles of naturopathic practice

Naturopathy is a traditional system of health care defined by core principles (see Fig. 1) and the use of an eclectic range of therapies that most commonly include dietary and lifestyle prescription, herbal medicines, and nutritional supplements [13]. Globally, naturopathic practitioners (NPs) provide care to diverse populations for a range of health conditions including, but not limited to, diseases identified as incurring a significant social and economic burden on societies [14]. The regulatory and legislative landscapes in which naturopathic practitioners provide care varies throughout the world, ranging from inclusion in national or state regulatory systems through to largely unregulated [15]. Equally, the use of naturopathy varies between 6.2 and 17.0% of the general populations studied to date, however this is based on limited prevalence research examining the naturopathic profession [16–18]. While differences in regulatory and legislative landscapes in different countries may affect the specific training and clinical practice behaviours of NPs based on their location [15, 19], the application of a core set of principles and philosophies is consistent throughout all world regions. Health promotion – defined as the process of enabling people to increase control over their health and its determinants, and thereby improve their health [20] - and patient education are reflected in the principles guiding practice for the naturopathic profession [21] and the application of these principles as an aspect of naturopathic practice is reported consistently by professional organisations around the world [22]. Furthermore, naturopathic practice approaches are reported to encourage positive health behaviours and self-care [23], possibly due to the emphasis NPs place on patient-centered care, health promotion and lifestyle counselling [24–26]. Yet the activities of NPs with regards to health promotion and community engagement beyond the clinical encounter have not been widely studied. In response, this paper provides the first international examination of health promotion behaviours of NPs. It describes NPs’ health promotion activities and explores the NP characteristics associated with undertaking commonly reported health promotion activities.

## Methods

### Design

A cross-sectional online survey of an international convenience sample of NPs.

### Ethical clearance

Ethical clearance was provided by the human research ethics committee of University of Technology Sydney (Approval no: ETH20–4725). Participants were provided with an information sheet which outlined the study aim, expected time required for survey completion, data storage processes, anonymity, and investigator team. Participant consent was implied through survey completion.

### Survey development and pretesting

The survey was developed by AS with input from IL on behalf of the World Naturopathic Federation (WNF) and informed by theoretical models and principles for health promotion [27] as well as previous national and international surveys of NPs’ practice behaviours [14, 28]. The survey was collaboratively drafted in English by both authors and tested for face validity by English-speaking naturopathic practitioners in Australia and Canada. The survey was then translated and reverse translated into Spanish, French, Portuguese and Slovene by native speakers of each language. All five language versions of the survey were uploaded to the survey platform SurveyGizmo and tested for language-specific face validity by one or two NPs for each language, following which minor edits to grammar and structure were applied to the survey before it was finalized for use.

### Survey instrument

The 15-item survey covered four domains: *demographics and practice characteristics*, *community education activities*, *community education topics and populations*, and *planning and designing community education activities*. The full survey, inclusive of participant information sheet, covered five pages or screens (see Supplementary File 1).

#### Demographics and practice characteristics

Participants were asked to identify their gender, country of naturopathic training and time since first naturopathic qualification. Respondents were also asked to indicate whether they were currently in clinical practice. Adaptive questioning was then applied to a further five items which were only seen by participants that indicated being in clinical practice. These items examined the country where they practice, their clinical practice environment, number of patients per week, number of patient hours per week, and the number of hours spent working on their business (i.e. not seeing patients) per week.

#### Community education activities

A survey item was presented to all participants which invited them to indicate the frequency (*daily, weekly, monthly, every few months, once or twice per year, less than once per year, never*) that they engaged in 23 different community education activities across four categories: *talks and presentations* (e.g. guest talks with community groups or patient-support groups), *communication through social and professional networks* (e.g. social media, blogs, email newsletters), i*nformation handouts* (e.g. handouts given to patients as part of the consultation), and *traditional media channels* (e.g. regular column in a newspaper or magazine).

#### Community education topics and populations

Participants were invited to select from a list of topics covered in community education activities (e.g. *self-care, preventing future health issues, naturopathic treatments*) and were also provided with a response option to provide a free text entry of ‘other’ topics. A similar list of populations targeted through community education activities (e.g. *general population, elderly, infants and children, disease-specific populations*) was also presented to participants, including a free text option to provide details regarding disease-specific populations. A further survey item asked participants to identify any additional clinical practice activities (e.g. *hospital visits, home visits, free consultations for specific populations*). Participants were able to select all relevant response options for each of these survey items.

#### Planning and designing community education activities

Two survey items examined participants’ approach to planning and designing community education activities. The first asked participants to attribute importance (*very, somewhat, not*) to four specific factors in their choice of topic for community education activities. The response options were designed based on the application of an established concept of *need*, as applied in the context of health promotion interventions [29]. The second item asked participant to identify the degree of stakeholder involvement (*very, somewhat, not at all*) during the development of community education activities. The response options were selected based on three *stakeholder types* relevant in health promotion interventions [29].

### Recruitment and sample

Participants were recruited via WNF Full Member Organisations; representing naturopathic professional associations in 35 countries. The WNF distributed an email to representatives of WNF full members that asked for an invitation to participate in the survey be shared with their individual members and their wider network of NPs within their jurisdiction. The survey link was also distributed via WNF social media platforms (i.e. Facebook, Twitter and Instagram). Access to the survey was open to any individual accessing the invitation to participate.

### Survey administration

The link to all survey translations were included in the email sent to all organisations via the World Naturopathic Federation with a request that the links be shared with their members through their usual communication channels. This may have included emails, e-newsletters, social media posts and others. The link directed users to the information sheet presented on the first page of the survey. Participation was voluntary and non-incentivised. Consent to participate was implied when participants progressed to the next page. Data were collected between 5th May and 1st July 2020. Participants were not forced to provide a response to any survey item, and no completeness checks were provided to the participants prior to survey submission.

### Response rates

In line with the ethical requirements of the study, no potentially identifiable information was collected from study participants including IP address. As such, participation rate was calculated as a proportion of the number of individuals who completed a survey item among those who accessed the survey information sheet. The completion rate was calculated as the proportion of individuals who completed at least one item related to community education activities (Screen #3 of survey). Differences in the demographic information among complete and incomplete survey responses was calculated based on responses to the demographic survey items (collected on Screen #2 of survey).

### Data cleaning

Responses from all five language versions were downloaded as a. CSV file and imported to Microsoft Excel (version 2006), merged into one dataset. The merged dataset was then imported to statistical analysis software (Stata 14.2). The survey items for each community education activity were recoded to encompass a binary variable (*did/did not use*) and a frequency variable for those who reported the activity (excluding those that did not use). These items were also collapsed into summary variables which categorized the individual community education activities into four new binary (yes/no) variables: *information sheets and handouts*, *information talks to community members*, *social and professional network communications*, and *traditional media channels*. Two further continuous variables were generated that presented a count of the number of individual community education activities and the number of community education activity categories reported by respondents. The two survey items reporting location by country were recoded to world regions. Additional binary categories (yes/no) were generated for the world regions where more than 100 completed responses were available for participants who indicated practicing in that world region. New variables were manually generated for disease population categories based on previous surveys of naturopathic practice [14, 28], and free text data regarding the disease-specific populations targeted through community education activities were coded to these categories.

### Data analysis

Descriptive analysis was conducted for all survey items, with frequencies and percentages calculated for categorical data and mean and standard deviation calculated for continuous data. The characteristics of individuals who did and did not complete the survey was compared using chi-square tests. The effect size for any statistically significant difference identified via the chi-square test was calculated using Cramer’s V, and was classified as negligible association (.00 and under .10); weak association (.10 and under .20); moderate association (.20 and under .40); relatively strong association (.40 and under .60); strong association (.60 and under .80) and very strong association (.80 and under 1.00), as reported by Rea and Parker (1992) [30].

A backwards stepwise regression analysis was performed to identify the most parsimonious line of practitioner characteristics (e.g. gender, time since first naturopathic qualification, clinical practice environment) and approach to designing community education activities (e.g. factors used to identify need when designing activities, stakeholders involved in activity design) most associated with a higher frequency of engaging in a selected community education activity. Four discrete models were generated through which the community education activity with the highest proportion of respondents from each category of activity (i.e., talks and presentations, communication through social networks, information handouts, traditional media channels) was selected as the model outcome. Each chosen activity used as a model outcome was converted to a binary variable (never = 0, all other frequency of use = 1). The potential predictors for each model were determined using chi square tests, whereby they were included in the baseline model if they were found to have an α value equal to or less than 0.2.

## Results

Of the 1038 individuals who accessed the survey, 906 provided any responses (participation rate = 87%). Of the individuals who responded, 813 completed survey items relevant to health promotion activities and were included in the analysis (completion rate = 89%). The world region where the respondent trained (Cramer’s V (V) = 0.32) or where they practice (V = 0.29) was moderately associated with completion status, while time since first qualification (V = 0.16) and whether the respondent was in clinical practice (V = 0.16) was weakly associated (see Supplementary File 2 for details). The results of the complete responses to the survey are presented below.

Table 1 presents the demographic characteristics of participants. They were predominantly female (77.5%) and represented NPs from all world regions. Approximately one third of participants (31.3%) received their first naturopathic qualification less than 5 years ago while 16.3% qualified more than 20 years ago. The majority (83.0%) of respondents reported currently being in clinical practice, of whom 38.8% were in clinical practice on their own and 22.9% were co-located with other health professionals but not other naturopaths. Those in clinical practice reported treating 19.5 patients (SD 44.9) across 18.2 h (SD 16.9) per week. An additional 16.9 h (SD 14.1) were reported for non-patient business activities per week. Over half of all participants reported either home visit consultations (30.3%) or free consultations for specific populations (23.1%).

**Table 1:** Participant characteristics

***Characteristic***	***N***	***%***
Gender (*n* = 813)		
*Female*	630	77.5
*Male*	179	22.0
*Non-binary*	4	0.5
World region where naturopathic training was completed (*n* = 801)		
*North American*	293	36.6
*Latin American*	72	9.0
*South East Asian*	28	3.5
*European*	130	16.2
*Western Pacific*	247	30.8
*Other (African, Eastern Mediterranean, not specified)*	31	3.9
Time since first naturopathic qualification (*n* = 811)		
*Less than 5 years*	254	31.3
*Between 5 and 10 years*	164	20.2
*Between 10 and 15 years*	161	19.9
*Between 15 and 20 years*	100	12.3
*More than 20 years*	132	16.3
Currently in clinical practice (*n* = 812)	674	83.0
World region where clinical practice is located (*n* = 668)		
*North American*	257	38.5
*Latin American*	57	8.5
*South East Asian*	22	3.3
*European*	117	17.5
*Western Pacific*	189	28.3
*Other (African, Eastern Mediterranean, not specified)*	26	3.9
Clinical practice environment (*n* = 672)		
*Solo clinic*	261	38.8
*Co-located with other health professionals but not other naturopaths*	154	22.9
*Co-located with other naturopaths but no other health professionals*	52	7.7
*Co-located with other naturopaths and other health professionals*	149	22.2
*Other clinical environment*	56	8.3
	***Mean***	***SD (min, max)***
Number of patients per week (*n* = 658)	19.5	44.9 (0, 1000)
Number of patient hours per week (*n* = 657)	18.2	16.9 (0, 130)
Number of hours working on their business per week (*n* = 657)	16.9	14.1 (1, 100)
Consultation types		
*Home visits (n = 814)*	247	30.3
*Free consultations for specific populations (n = 814)*	188	23.1
*Free health screening services (*e.g. *blood pressure checks) (n = 814)*	80	9.8
*Hospital visits (n = 814)*	51	6.3

### Health promotion activities

Almost all participants (98%) reported at least one health promotion activity, most reported were information sheets and handouts (92.7%) or social and professional network communications (91.8%). Information talks presented to community members were also frequently reported (84.9%) while traditional media channels were reported less frequently, although still by more than half (52.8%) of respondents. Participants also reported community education engagements across approximately three different categories (mean 3.2; SD 0.92; min 0, max 4) and between nine and ten individual activities (mean 9.5; SD 4.7; min 0, max 23).

Further details of the health promotion activities undertaken by participants are presented in Table 2. A substantial proportion of participants reported giving either individualised (84.5%) or pre-prepared information handouts (84.5%) directly to patients as part of consultations, or using social media (84.6%), to educate the community. These activities were also reported as employed daily, weekly, or monthly by most users. Guest talks with community or patient-support groups (no fee charged to attendees) were also reported by many participants (72.4%) but were more commonly reported to occur every few months or less. Of the traditional media channels, more participants reported contributing invited expert comments for newspaper and magazine articles (41.1%) than other forms, with most of those respondents indicating this occurred less than once per year (35.8%).

**Table 2:** Frequency of use of health promotion activities

Health promotion activity	Yes	Frequency					
		***Daily***	***Weekly***	***Monthly***	***Every few months***	***Once or twice per year***	***Less than once per year***
	*N (%)*	*N (%)*	*N (%)*	*N (%)*	*N (%)*	*N (%)*	*N (%)*
Talks and presentations							
*Guest talks with community or patient-support groups (no fee charged to attendees) (n = 739)*	535 (72.4)	33 (6.2)	47 (8.8)	70 (13.1)	132 (24.7)	136 (25.4)	117 (21.9)
*Guest talks with community or patient-support groups (fee charged to attendees) (n = 732)*	412 (56.3)	37 (9.0)	40 (9.7)	49 (11.9)	78 (18.9)	109 (26.5)	99 (24.0)
*Talks presented to the community and held within your clinic (no fee charged to attendees) (n = 728)*	388 (53.3)	14 (3.6)	26 (6.7)	46 (11.9)	78 (20.1)	108 (27.8)	116 (29.9)
*Talks presented to the community and held within your clinic (fee charged to attendees) (n = 724)*	290 (40.1)	14 (4.8)	24 (8.3)	38 (13.1)	61 (21.0)	77 (26.6)	76 (26.2)
*Online seminars or workshops (no fee charged to attendees) (n = 716)*	301 (42.0)	13 (4.3)	23 (7.6)	62 (20.6)	73 (24.3)	64 (21.3)	66 (21.9)
*Online seminars or workshops (fee charged to attendees) (n = 708)*	268 (37.9)	7 (2.6)	30 (11.2)	33 (12.3)	58 (21.6)	61 (22.8)	79 (29.5)
Communication through social and professional networks							
*Social media (*e.g. *Facebook, Instagram, Twitter) (n = 728)*	616 (84.6)	169 (27.4)	219 (35.6)	99 (16.1)	71 (11.5)	32 (5.2)	26 (4.2)
*Blogs (n = 725)*	422 (58.2)	24 (5.7)	85 (20.1)	113 (26.8)	103 (24.4)	57 (13.5)	40 (9.5)
*Email newsletter (n = 722)*	418 (57.9)	14 (3.4)	45 (10.8)	137 (32.8)	103 (24.6)	63 (15.1)	56 (13.4)
*Vlog (*e.g. *YouTube channel) (n = 718)*	208 (29.0)	10 (4.8)	32 (15.4)	37 (17.8)	55 (26.4)	28 (13.5)	46 (22.1)
*Invited expert comment on a podcast (n = 722)*	160 (22.2)	1 (0.6)	10 (6.3)	14 (8.8)	33 (20.6)	37 (23.1)	65 (40.6)
*Print newsletter (n = 719)*	136 (18.9)	7 (5.2)	10 (7.4)	28 (20.6)	21 (15.4)	30 (22.1)	40 (29.4)
*Regular segment on a podcast (n = 720)*	72 (10.0)	2 (2.8)	11 (15.3)	13 (18.1)	12 (16.7)	15 (20.8)	19 (26.4)
Information handouts							
*Individualised handouts given directly to patients as part of the consultation (n = 729)*	616 (84.5)	334 (54.2)	150 (24.4)	60 (9.7)	39 (6.3)	13 (2.1)	20 (3.3)
*Pre-prepared handouts given directly to patients as part of the consultation (n = 722)*	588 (81.4)	245 (41.7)	181 (30.8)	63 (10.7)	56 (9.5)	23 (3.9)	20 (3.4)
*Information handouts in the clinic waiting room (n = 729)*	502 (68.9)	181 (36.1)	70 (13.9)	84 (16.7)	71 (14.1)	47 (9.4)	49 (9.8)
*Information handouts available for download from your website (n = 723)*	285 (39.4)	93 (32.6)	36 (12.6)	57 (20.0)	46 (16.1)	20 (7.0)	33 (11.6)
Traditional media channels							
*Invited expert comment for newspaper or magazine articles (n = 721)*	296 (41.1)	7 (2.4)	15 (5.1)	28 (9.5)	65 (22.0)	75 (25.3)	106 (35.8)
*Regular column in newspaper or magazine (n = 720)*	135 (18.8)	4 (3.0)	8 (6.0)	30 (22.2)	23 (17.1)	19 (14.1)	51 (37.8)
*Invited expert comment on a radio program (n = 722)*	209 (29.0)	2 (1.0)	11 (5.3)	16 (7.7)	30 (14.4)	45 (21.5)	105 (50.2)
*Regular segment on a radio program (n = 720)*	87 (12.1)	4 (4.6)	11 (12.6)	10 (11.5)	15 (17.2)	15 (17.2)	32 (36.8)
*Invited expert comment on a television program (n = 723)*	124 (17.2)	1 (0.8)	7 (5.7)	5 (4.0)	17 (13.7)	22 (17.7)	72 (58.1)
*Regular segment on a television program (n = 716)*	38 (5.3)	2 (5.3)	5 (13.2)	3 (7.9)	9 (23.7)	4 (10.5)	15 (39.5)

### Health promotion topics and target populations

The topics covered by participants’ health promotion activities included effective ways to change health behaviours for improved health (69.9%), self-care (69.3%), managing current health issues (65.6%), and preventing future health issues (65.5%) (see Table 3). The activities were mostly aimed at the general population (77.8%) although a number of participants also reported targeting populations based on sociodemographic factors such as life stage (infants and children [23.7%], elderly [21.3%]) or income level (low income [21.5%]). Health promotion activities were reported as being disease-specific by 22.7% of participants. The topic focus for these activities was most reported as endocrine (25.4%) and autoimmune or allergy conditions (21.1%).

**Table 3:** Topics discussed and populations targeted in health promotion activities (*n* = 814)

Topics	
*Naturopathic approaches to understanding health*	587 (72.1)
*Effective ways to change health behaviours for improved health*	569 (69.9)
*Self-care*	564 (69.3)
*Managing current health issues*	534 (65.6)
*Preventing future health issues*	533 (65.5)
*Naturopathic treatments*	500 (61.4)
*Causes of ill health*	495 (60.8)
*Naturopathic principles and philosophies*	462 (56.8)
Populations targeted	
*General population*	633 (77.8)
*Individuals with low income*	175 (21.5)
*Infants and children*	193 (23.7)
*Pregnant women*	166 (20.4)
*Elderly*	173 (21.3)
*Military personnel or veterans*	35 (4.3)
*Disease-specific populations*	185 (22.7)
*Disease-specific topics:*^a^	
Endocrine	47 (25.4)
Autoimmune and allergy	39 (21.1)
Cancer	34 (18.4)
Mental health	32 (17.3)
Female reproductive	31 (16.8)
Musculoskeletal	26 (14.1)
Cardiovascular	26 (14.1)
Gastrointestinal	19 (10.3)
Neurological	15 (8.1)
Weight management	14 (7.6)
Maternal health	*9 (4.9)*
Skin conditions	*7 (3.8)*
Infectious disease	*7 (3.8)*
Ageing and cognition	*6 (3.2)*
Urogenital and men’s health	*5 (2.7)*
Respiratory	*1 (0.5)*

^a^Percentages calculated from number of respondents indicating they targeted disease-specific populations

### Identifying need and involving stakeholders in the design of health promotion activities

Most participants indicated that the health issues that individuals in their community said they need help with (79.5%) and expert advice and evidence about the health issues affecting the community (77.4%) were very important considerations when identifying the need for their health promotion activities (see Table 4). Participants most frequently reported all three stakeholder categories as ‘somewhat involved’ in the design of a health promotion activity. Individuals who the participant expected will benefit from the activity were most frequently reported as ‘very involved’ in design of the activity (43.2%).

**Table 4:** Identifying need and involving stakeholder to inform the design of health promotion activities

**Identification of need when designing activities**	**Very important**	**Somewhat important**	**Not important**
*Expert advice and evidence about the health issues affecting the community (n = 672)*	520 (77.4)	138 (20.5)	14 (2.1)
*The health issues affecting your community compared to other populations (n = 661)*	370 (56.0)	244 (36.9)	47 (7.1)
*The health issues individuals in your community have said they need help with (n = 674)*	536 (79.5)	125 (18.6)	13 (1.9)
*The health issues you have identified based on the types of health services available to your community (n = 663)*	461 (69.5)	183 (27.6)	19 (2.9)
**Stakeholder involvement in activity design**	**Very involved**	**Somewhat involved**	**Not at all involved**
*Individuals who you expect will benefit from the activity (n = 658)*	284 (43.2)	290 (44.1)	84 (12.8)
*Individuals involved in the delivery of the activity (n = 652)*	224 (34.4)	313 (48.0)	115 (17.6)
*Individuals without whom the activity would not be possible (n = 652)*	246 (37.7)	279 (42.8)	127 (19.5)

Table 5 presents the results of the backwards stepwise logistic regression analysis, identifying the characteristics associated with a higher frequency of use of the most common health promotion activity for each category. Males were more likely to report providing guest talks to community or patient-support groups for no charge (odds ratio [OR] 1.67, *p* = 0.04) and invited expert comment to newspaper and magazine articles (OR 1.50, *p* = 0.04), compared to females. Practitioners who had received their first naturopathic qualification more than 5 years ago were less likely to report using social media to communicate health messages to the public (e.g., more than 20 years: OR 0.37, *p* = 0.005) but more likely to be invited to provide expert comment for newspaper or magazine articles (e.g., more than 20 years: OR 1.84, *p* = 0.02) compared to practitioners who qualified less than 5 years ago. Participants who considered expert advice and evidence about health issues affecting the community to be ‘not important’ (OR 0.12, *p* = 0.001), rather than ‘very important’, when designing activities were less likely to provide guest talks to community or patient-support groups for no charge. Similarly, participants who perceived the health issues affecting their community compared to other populations as ‘somewhat important’, rather than ‘very important’, in their design of community education activities were less likely to provide expert comment to newspapers or magazines (OR 0.65, *p* = 0.04). Participants who identified that the health issues individuals in their community have said they need help with as ‘not important’, rather than ‘very important’, to consider when designing an activity were less likely to report using social media to communicate with the public (OR 16, *p* = 0.004) or to provide individualised handouts to patients during consultation (OR 0.17, *p* = 0.02). In terms of stakeholder involvement, participants that indicated individuals involved in the delivery of the activity were ‘somewhat involved’ (RR 0.82, *p* = 0.001) or ‘not at all involved’ (RR 0.73, *p* = 0.001), rather than ‘very involved’, in the design of an activity were less likely to report giving guest talks to community or patient-support groups for no charge. Compared to participants who identified individuals expected to benefit from the activity as ‘very involved’, participants who identified such individual as ‘somewhat involved’ (OR 0.64, p0.01) were less likely to provide invited expert comment to a newspaper or magazine. Similarly, those who identified such individuals as ‘not at all involved’ (OR 0.42, *p* = 0.01) were less likely to use social media.

**Table 5:** Results of backwards stepwise logistic regression analysis to describe the factors associated with the most common health promotion activities within each category

Characteristics	*Health promotion activity*							
	*Guest talks with community or patient-support groups (no fee charged to attendees)*^a^		*Social media (*e.g.*, Facebook, Instagram, Twitter)*^b^		*Invited expert comment for newspaper or magazine articles*^c^		*Individualised handouts given directly to patients as part of the consultation*^d^	
	Odds ratio (95% CI)	***p***	Odds ratio (95% CI)	***p***	Odds ratio (95% CI)	***p***	Odds ratio (95% CI)	***p***
Gender^a^								
*Female*	Ref	0.04	–	*–*	*Ref*	0*.*04	–	–
*Male*	1.67 (1.02–2.71)		–	*–*	1.50 (1.02–2.21)		–	–
Time since first naturopathic qualification								
*Less than 5 years*	–	*–*	Ref	–	Ref	*–*	–	–
*Between 5 and 10 years*	–	*–*	0.62 (0.31–1.24)	0.18	1.22 (0.77–1.93)	*0.41*	–	–
*Between 10 and 15 years*	–	*–*	0.59 (0.29–1.18)	0.05	1.92 (1.21–3.03)	*0.005*	–	–
*Between 15 and 20 years*	–	*–*	0.57 (0.26–1.27)	0.25	2.68 (1.54–4.65)	*< 0.001*	–	–
*More than 20 years*	–	*–*	0.37 (0.18–0.74)	0.005	1.84 (1.09–3.10)	*0.02*	–	–
Identification of need when designing activities								
*Expert advice and evidence about the health issues affecting the community*	–	*–*	–	*–*	–	*–*		
*Very important*	Ref	–	–	–	Ref	–	–	–
*Somewhat important*	0.84 (0.53–1.32)	0.44	–	*–*	0.65 (0.43–0.98)	*0.04*	–	–
*Not important*	0.12 (0.0.-0.44)	0.001	–	*–*	0.37 (0.10–1.40)	*0.15*	–	–
*The health issues affecting your community compared to other population*								
*Very important*	–	–			Ref	–	–	–
*Somewhat important*	–	–			0.85 (0.75–0.96)	0.007	–	–
*Not important*	–	–			0.86 (0.68–1.09)	0.2	–	–
*The health issues individuals in your community have said they need help with*								
*Very important*	–	–	Ref	–	–	–	Ref	
*Somewhat important*	–	–	0.81 (0.46–1.41)	0.46	–	*–*	0.48 (0.27–0.84)	*0.01*
*Not important*	–	–	0.16 (0.05–0.55)	0.004	–	*–*	0.17 (0.04–0.70)	*0.02*
Stakeholder involvement in activity design			–	–	–	–		
*Individuals involved in the delivery of the activity*								
*Very involved*	Ref		–				–	–
*Somewhat involved*	0.53 (0.33–0.84)	0.007	–	–	–	–	–	–
*Not at all involved*	0.24 (0.14–0.42)	< 0.001	–	–	–	–	–	–
*Individuals who you expect will benefit from the activity*								
*Very involved*			Ref	–	Ref		–	–
*Somewhat involved*			0.86 (0.52–1.45)	0.58	0.64 (0.45–0.91)	0.01	–	–
*Not at all involved*			0.42 (0.22–0.82)	0.01	0.61 (0.36–1.04)	0.07	–	–

^a^*Predictors included in the baseline model (group talks)*: gender; expert advice and evidence about the health issues affecting the community; the health issues individuals in your community have said they need help with; the health issues you have identified based on the types of health services available to your community; individuals who you expect will benefit from the activity; individuals involved in the delivery of the activity; individuals without whom the activity would not be possible

^b^*Predictors included in the baseline model (social media)*: expert advice and evidence about the health issues affecting the community; the health issues individuals in your community have said they need help with; individuals who you expect will benefit from the activity; individuals involved in the delivery of the activity; individuals without whom the activity would not be possible

^c^*Predictors included in the baseline model (expert comment)*: gender; expert advice and evidence about the health issues affecting the community; the health issues individuals in your community have said they need help with; the health issues you have identified based on the types of health services available to your community; individuals who you expect will benefit from the activity; individuals involved in the delivery of the activity; individuals without whom the activity would not be possible

^d^*Predictors included in the baseline model (individualised handouts)*: expert advice and evidence about the health issues affecting the community; the health issues individuals in your community have said they need help with; the health issues you have identified based on the types of health services available to your community

## Discussion

### NPs engage in community education and health promotion activities

This study is the first known examination of health promotion activities undertaken by NPs and presents several important findings. Firstly, the study findings indicate that NPs engage in activities aimed at educating the community. One reason for the extent to which NPs appear to engage with health promotion and community education is the alignment between these activities and the guiding naturopathic principles [21], which positions health promotion as central to naturopathic practice. In contrast, other primary care practitioners (i.e. general practitioners and nurses) commonly perceive health promotion activities as educational tasks that are the responsibility of the community or government and therefore is peripheral to their own field of work [31]. This avoidance of health promotion activities among primary care professions has been linked to the biomedical perspective which de-emphasises social determinants of health, illness prevention and promotion of healthy lifestyles [31]. Health promotion interventions carried out in primary care settings have historically focused on reducing cardiovascular risk factors, encouraging physical activity, and improving self-care in individual with chronic illness [32]. These topics are all reflected in the topics discussed by the NPs included in our study, however it is notable that other topics such as naturopathic approaches to understanding health and naturopathic treatments were also commonly reported and are likely unique to naturopathic practice. Despite these differences, our study suggests NPs are engaging in health promotion activities and as such their potential impact on community health should be examined within the broader context of health promotion in primary care practice.

### NPs employ diverse communication methods to educate the community

Our study suggests that NPs not only engage with community education and health promotion, but that they employ diverse communication methods including talks and presentations, social and professional networks, information handouts and traditional media channels. This aligns with recommended health communication practices that advise using a range of communication channels to provide health information to the community [11]. Furthermore, the diversity of education methods employed by NPs matches contemporary research regarding health communication which has shown that successful modification of health behaviours in the community targets specific populations and employs multiple communication activities and channels [12]. Given one of the most common topics reported by our study participants related to changing health behaviours to improve health, the varied approaches employed by NPs to educate individuals in their community may improve the success of their efforts. Furthermore, individuals who visit with a NP may be more motivated to engage in positive health behaviours [33] and as such, the community education activities undertaken by NPs may have particularly marked impact in their patient population compared to other members of the community. However, any health communication and community education strategies must be evaluated to garner feedback from the target population and assess the overall effectiveness of any activity in achieving its stated goal [11, 12]. This is particularly important in the context of our study findings given the variation with which participants appeared to consider the health issues in the community when designing community education activities. Unfortunately, our research does not provide any insights into the evaluation or feedback methods employed by NPs or the outcomes of their activities and future research should address this gap.

### NPs commonly used social media to educate the community

The community education delivery method most reported by NPs was social media, while traditional broadcast media channels (newspaper, magazines, radio, television) were reported much less frequently. This is an interesting finding as social media provides an intersect between information from online sources and reliance on personal social networks [9]. Social media is used for health communication for several reasons including: increasing interactions with others; providing more available, shared and tailored information; increasing accessibility and widening access to health information; and providing peer, social or emotional support [9]. It has also been flagged as a potentially useful, and largely untapped, tool in health promotion and education to encourage individuals to modify their health behaviours [34]. These features of social media align with other findings in our study including the propensity for NPs to prioritise reach and access to their community education activities by preferencing free rather than charged information talks, and otherwise using communication channels that are accessible to a broad section of the community. These findings also reflect existing research which indicates individuals are influenced by information from family and friends when making health-care decisions regarding the use of products and practices such as naturopathy, and rely much less on information from traditional media channels [35]. The degree to which the populations intended by NPs as target audiences are reached by social media campaigns is unclear, however, and may depend on the specific platforms being employed. Survey research has described the differentiated use of social media by patients, based upon which Twitter is reportedly used most often for communicating knowledge-based information and Facebook for social support, while both platforms are used for exchanging advice [8]. However, health communicators have also raised concerns regarding the lack of reliability, confidentiality and privacy of information shared via social media [9] and these concerns are also held by patients [8]. In contrast, health professionals identify a lack of skills as the primary barrier to their use of social media [8]. While our study identifies a substantial proportion of NPs use social media to educate the community, their self-perceived social media competence is unknown. However, our analysis did find that they were less likely to use social media if they had been in practice for more than 5 years, and the trend to decreased likelihood had a direct relationship with a greater number of years since first qualifying (e.g., practitioners that qualified more than 20 years ago were least likely to use social media). The reason behind this pattern is not known but may be due to practitioners using social media to raise awareness about their health services as they establish their practice after graduation. Alternatively, it may be due to their age with more recent graduates likely to be younger and therefore more familiar with using social media. However, it is important to acknowledge that naturopathic programs are recognised for having non-typical student cohorts characterised in part by a greater proportion of mature-age students transitioning to a new career [36] and an often complex relationship with technology use [37, 38]. As such a relationship between recency of graduation, graduate age and technology behaviours should not be assumed in this case. Equally, the effectiveness of their social media community education activities is unclear, and may benefit from careful evaluation and integration with wider, comprehensive community education campaigns [39]. Other forms of social and professional network communication tools such as blogs and patient newsletters were reported to be used by many NPs and these forms of communication may provide a more targeted form of education to a specific patient population.

### NPs incorporate education activities into their clinical consultations

Our study indicates NPs are providing information directly to individuals either during clinical consultations or through presentations to community or patient-support groups. Various types of information handouts, for example, were a common delivery method used by NPs to educate the community, primarily within their clinical practice. For these materials to be effective, they must be written at a level able to be read and understood by users and as such the patient’s health literacy as well as the readability and design of the handout must be carefully considered [40]. However, previous research investigating the quality of information handouts in health service delivery suggests insufficient attention may be given to the suitability and usability of information handouts currently given to patients by the health professionals providing their care [41, 42]. Certainly, while NPs providing individualised handouts to their patients were more likely to give importance to patient-identified health needs, our study findings also suggest individuals intended to benefit from the intervention are ‘very involved’ in designing a NPs’ community education activity in less than half of cases and as such there is potential for missed opportunities due to poor engagement with end users when designing resources such as handouts. However, providing patients with handouts that are individualised to their specific needs and delivered alongside verbal information – a practice reported by more than 80% of NPs - can notably improve patient’s knowledge [43] and may offset the lower level of user engagement during the design process. NPs also reported outreach health promotion activities, whereby they delivered information to community or patient-support groups and, most commonly, did not charge for attendance. Outreach has historically been used to reduce health inequalities by improving access to health services and information, and encountering groups of people who might not have otherwise visited a clinical setting [7]. Given the importance placed on health education to improve health equity and literacy in vulnerable or marginalised populations [4, 5], the findings of this study suggest NPs are volunteering their time to actively provide health education to the community and this finding warrants closer attention from health policy makers.

### NPs provide education on diverse topics to a variety of populations

The NPs participating in this study reported covering diverse topics and targeting a variety of populations through their community education activities. This diversity reflects existing international research documenting the types of clients consulting with a NP and the health concerns for which they are seeking naturopathic care [14]. The conditions reported most frequently by respondents also represent non-communicable diseases that significantly impact the global burden of diseases [44]. Patient education as part of clinical care is a crucial element to the prevention and treatment of non-communicable diseases such as type 2 diabetes, and can improve clinical, lifestyle and psycho-social outcomes [45]. Given endocrine conditions was the disease category most reported by NPs providing education on disease-specific topics, the value and benefit of NP-delivered education for individuals with endocrine conditions, such as type 2 diabetes, should be explored further. Retrospective analysis of naturopathic clinical records has found NPs consistently provide dietary counselling, prescribe exercise and teach stress reduction techniques to individuals with type 2 diabetes [46]. Furthermore, patients with type 2 diabetes had improved haemoglobin-A1C levels compared to usual care, and reported improved dietary intake and physical activity, 6 months after initiating consultations with a NP [23]. Glucose self-monitoring, mood, self-efficacy and motivation to change their lifestyle was also improved for these individuals six and 12 months after initiating treatment. The reason for these positive changes to health behaviour may be linked to the patients experiencing their interaction with their NP as patient-centred, collaborative and holistic rather than disease-focused [24]. Furthermore, the NP clinical encounter is described by individuals with type 2 diabetes as characterised by: individualised and detailed health promotion; counselling that promotes self-efficacy; encompassing pragmatic and practical self-care recommendations; and providing information that addressed both diabetes self-care and general health [24]. While this previous research relates specifically to type 2 diabetes, other research reports a similar emphasis on patient-centredness, empowerment and patient education by NPs when providing care to individuals diagnosed with health conditions such as cancer [47] and other chronic illnesses [25, 26]. In addition to examining the clinical effect of naturopathic approaches to patient education, it is also worth exploring the specific content of the information shared by NPs with patients and the community as naturopathy encourages a holistic understanding of health which includes an emphasis on interaction between physiological systems [48].

## Limitations

This study is not without limitations. While this is the first international study to date examining health promotion activities of NPs, we do not know the number of NPs who were sent the invitation and as such are unable to calculate a true response rate for our study. For this reason, the findings may not be generalisable to the aggregate international naturopathic profession. However, the preliminary findings from this study indicate that additional more focused studies would be worthwhile. The diversity of naturopathic practice in specific geographical areas is likely to be impacted by the professional formational status in each country as well as, cultural, social, technological and regulatory influences and this requires consideration within the context of these national and regional settings. Additional bias may have been introduced by the self-reported nature of the survey data as the accuracy of this data was not independently confirmed by the researchers. The target population was limited to full members (national professional associations) of the WNF and therefore biases may have been introduced by excluding naturopaths who possibly have lower standards of professional practice, particularly in countries were regulatory mechanisms ensuring consistency in training and practice are absent. Further, the study may be subject to responder bias in that naturopathic practitioners engaged with health promotion activities or more interested in engaging with research may have been more likely to participate. Regardless of these limitations, this study provides an important contribution to the understanding of naturopathic health promotion and community education behaviours and activities at an international level.

## Conclusions

 Health promotionis a key activity of the global naturopathic profession. There are a wide range of patient education tools utilized by NPs with some variance between the specific methods used depending upon characteristics of the NP. Health promotion and patient education are widely acknowledged by health policy makers and public health practitioners as crucial ingredients to successfully improving the health of a community. For this reason, a closer examination of the specific health promotion activities undertaken by NPs, including the information conveyed to the public and the impact of that information on health behaviour, is urgently required. The findings of this study, and future research emerging from this exploratory work, may have significant value to all parties committed to improved primary care, public health and health promotion efforts internationally.

## Supplementary Information

**Additional file 1.**

**Additional file 2: Supplementary Table 2.** Comparison of demographic characteristics between complete and incomplete responders to survey.

## Data Availability

The datasets used and/or analysed during the current study are available from the corresponding author on reasonable request.

## Abbreviations

NPs: Naturopathic practitioners
WNF: World Naturopathic Federation
