# Supplementary material for: Community education and health promotion activities of naturopathic practitioners: results of an international cross-sectional survey
Source: BMC Complement Med Ther. 2021 Nov 30;21:293. doi: 10.1186/s12906-021-03467-z (PMC8630897; doi:10.1186/s12906-021-03467-z)
Supplement: Supplementary file 1 — Additional file 1. [file 12906_2021_3467_MOESM1_ESM.docx]

# An International Study of Health Promotion and Community Education Activities of Naturopathic Clinicians

## About this study

ID: 2

### PARTICIPANT INFORMATION SHEET An International Study of Health Promotion and Community Education Activities of Naturopathic Clinicians (#XXXX) WHO IS DOING THE RESEARCH? My name is Dr Amie Steel and I am an academic at University of Technology Sydney WHAT IS THIS RESEARCH ABOUT? This research is to find out about health promotion and community education activities undertaken by naturopathic clinicians FUNDING No funding has been received for this project. WHY HAVE I BEEN ASKED? You have been invited to participate in this study because you are a naturopath who is a member of a professional association recognised by the World Naturopathic Federation.  Your association has been asked by the World Naturopathic Federation to pass this invitation on to you on behalf of Dr Steel.   IF I SAY YES, WHAT WILL IT INVOLVE? If you decide to participate, I will invite you to complete a short online survey about any activities you may undertake to promote health in the community through education. The survey will take approximately ten (10) minutes to complete. ARE THERE ANY RISKS/INCONVENIENCE? Yes, there are some risks/inconvenience.  This study requires access to the internet and as such any barriers to reliable internet access in your country may cause some inconvenience to study participation.   DO I HAVE TO SAY YES? Participation in this study is voluntary. It is completely up to you whether or not you decide to take part. WHAT WILL HAPPEN IF I SAY NO? If you decide not to participate, it will not affect your relationship with the researchers, the University of Technology Sydney, your professional association or the World Naturopathic Federation. However, as the survey is anonymous it is not possible to withdraw from this study once you have begun the survey. CONFIDENTIALITY By beginning the survey you are demonstrating consent to participate in this study. The research team will collect information about you for this project and all of this information will be treated confidentially. As the survey is anonymous your confidentiality is protected. Your responses will only be used for the purposes of this research project. We plan to publish the results in a peer-reviewed journal publication for wide public dissemination. WHAT IF I HAVE CONCERNS OR A COMPLAINT? If you have concerns about the research that you think I can help you with, please feel free to contact me on amie.steel@uts.edu.au. If you would like to verify my identity please contact Dr Iva Lloyd, President of the World Naturopathic Federation on president@worldnaturopthicfederation.org   NOTE:  This study has been approved in line with the University of Technology Sydney Human Research Ethics Committee [UTS HREC] guidelines.  If you have any concerns or complaints about any aspect of the conduct of this research, please contact the Ethics Secretariat on ph.: +61 2 9514 2478 or email: Research.Ethics@uts.edu.au], and quote the UTS HREC reference number (#XXXX).  Any matter raised will be treated confidentially, investigated and you will be informed of the outcome.

## About you

ID: 3

#### 1) What is your gender?

( ) Female

( ) Male

( ) Non-binary

ID: 4

#### 2) How long ago did you first qualify as a naturopathic clinician?

( ) Less than 5 years

( ) Between 5 and 10 years

( ) Between 10 and 15 years

( ) Between 15 and 20 years

( ) More than 20 years

**Logic: Show/hide trigger exists.**

ID: 5

#### 3) Are you currently in clinical practice?

( ) Yes

( ) No

Validation: Must be numeric Whole numbers only Positive numbers only

**Logic: Hidden unless: #3 Question "Are you currently in clinical practice?" is one of the following answers ("Yes")**

ID: 6

### 4) On average, how many patients consult with you in your clinical practice each week?

_________________________________________________

**Logic: Hidden unless: #3 Question "Are you currently in clinical practice?" is one of the following answers ("Yes")**

ID: 7

#### 5) Which of the following best describes your clinical practice environment?

( ) I am in a clinic by myself

( ) I am in a clinic with other health professionals but no other naturopaths

( ) I am in a clinic with other naturopaths but no other types of health professionals

( ) I am in in a clinic with other naturopaths and other health professionals

( ) Other (please give details): _________________________________________________*

## Health Promotion and Community Education Activities

ID: 20

#### 6) How often do you use the following activities to promote health and educate the general population? Do not include activities designed to specifically target health professionals or other colleagues

|  | **Never** | **Daily** | **Weekly** | **Monthly** | **Every few months** | **Once or twice per year** | **Less than once per year** |
| --- | --- | --- | --- | --- | --- | --- | --- |
| Guest talks with community groups or patient-support groups | ( ) | ( ) | ( ) | ( ) | ( ) | ( ) | ( ) |
| Talks presented to the community held in your clinic | ( ) | ( ) | ( ) | ( ) | ( ) | ( ) | ( ) |
| Online seminars or workshops | ( ) | ( ) | ( ) | ( ) | ( ) | ( ) | ( ) |
| Blogs | ( ) | ( ) | ( ) | ( ) | ( ) | ( ) | ( ) |
| Vlog (e.g. YouTube channel) | ( ) | ( ) | ( ) | ( ) | ( ) | ( ) | ( ) |
| Email newsletter | ( ) | ( ) | ( ) | ( ) | ( ) | ( ) | ( ) |
| Print newsletter | ( ) | ( ) | ( ) | ( ) | ( ) | ( ) | ( ) |
| Social media (e.g. Facebook, Instagram, Twitter) | ( ) | ( ) | ( ) | ( ) | ( ) | ( ) | ( ) |
| Information handouts in the clinic waiting room | ( ) | ( ) | ( ) | ( ) | ( ) | ( ) | ( ) |
| Pre-prepared handouts given directly to patients as part of the consultation | ( ) | ( ) | ( ) | ( ) | ( ) | ( ) | ( ) |
| Individual handouts tailored to the individual given directly to patients as part of the consultation | ( ) | ( ) | ( ) | ( ) | ( ) | ( ) | ( ) |
| Information handouts available for download from a website | ( ) | ( ) | ( ) | ( ) | ( ) | ( ) | ( ) |
| Invited expert comment for newspaper or magazine articles | ( ) | ( ) | ( ) | ( ) | ( ) | ( ) | ( ) |
| Regular column in newspaper or magazine | ( ) | ( ) | ( ) | ( ) | ( ) | ( ) | ( ) |
| Invited expert comment on a radio program | ( ) | ( ) | ( ) | ( ) | ( ) | ( ) | ( ) |
| Regular segment on a radio program | ( ) | ( ) | ( ) | ( ) | ( ) | ( ) | ( ) |
| Invited expert comment on a television program | ( ) | ( ) | ( ) | ( ) | ( ) | ( ) | ( ) |
| Regular segment on a television program | ( ) | ( ) | ( ) | ( ) | ( ) | ( ) | ( ) |

## Health Promotion and Community Education Topics and Populations

ID: 35

#### 7) What topics do you cover in your health promotion and community education activities? (please select all that apply)

[ ] Self-care

[ ] Causes of ill health

[ ] Effective ways to change health behaviours for improved health

[ ] Preventing future health issues

[ ] Managing current health issues

[ ] Naturopathic approaches to understanding health

[ ] Naturopathic treatments

[ ] Naturopathic principles and philosophies

[ ] Other (please give details): _________________________________________________*

[ ] None of the above

ID: 36

#### 8) What types of populations do you try to support through your health promotion and community education activities?

[ ] General population

[ ] Elderly

[ ] Disease-specific populations (please give details)

[ ] Infants and children

[ ] Individuals with low income

[ ] Military personnel or veterans

[ ] Pregnant women

[ ] Other (please give details): _________________________________________________*

[ ] None of the above

**Logic: Hidden unless: #3 Question "Are you currently in clinical practice?" is one of the following answers ("Yes")**

ID: 37

#### 9) What types of additional clinical practice activities do you undertake to support your patients? (Please select all that apply)

[ ] Hospital visits

[ ] Home visits

[ ] Free consultations for specific populations

[ ] Free consultations or treatments for specific populations (please give details): _________________________________________________*

[ ] None of the above

## Planning and designing health promotion and community education activities

ID: 8

#### 10) How important are the following factors in your choice of topic for your health promotion and community education activities?

|  | **Very important** | **Somewhat important** | **Not at all important** |
| --- | --- | --- | --- |
| Expert advice and evidence about the health issues affecting the community | ( ) | ( ) | ( ) |
| The health issues affecting your community compared to other populations | ( ) | ( ) | ( ) |
| The health issues individuals in your community have said they need help with | ( ) | ( ) | ( ) |
| The health issues you have identified based on the types of health services and treatments used by individuals in your community | ( ) | ( ) | ( ) |

ID: 13

#### 11) How involved are the following groups in the development of your health promotion and community education activities?

|  | **Very involved** | **Somewhat involved** | **Not at all involved** |
| --- | --- | --- | --- |
| Individuals who you expect will benefit from the activity | ( ) | ( ) | ( ) |
| Individuals involved in the delivery of the activity | ( ) | ( ) | ( ) |
| Individuals without whom the activity would not be possible | ( ) | ( ) | ( ) |

ID: 47

### 12) Please provide any additional details regarding your health promotion or community education activities that you would like to share

____________________________________________

____________________________________________

____________________________________________

____________________________________________

## Thank You!

ID: 1

### Thank you for taking this survey.
