# Supplementary material for: Community education and health promotion activities of naturopathic practitioners: results of an international cross-sectional survey
Source: BMC Complement Med Ther. 2021 Nov 30;21:293. doi: 10.1186/s12906-021-03467-z (PMC8630897; doi:10.1186/s12906-021-03467-z)
Supplement: Supplementary file 2 — Additional file 2: Supplementary Table 2. Comparison of demographic characteristics between complete and incomplete responders to survey. [file 12906_2021_3467_MOESM2_ESM.docx]

Supplementary Table 2: Comparison of demographic characteristics between complete and incomplete responders to survey

| Characteristic | Complete responders (n=814) | Incomplete responders  (n=92) | *p* | V* |
| --- | --- | --- | --- | --- |
|  | N (%) | N (%) |  |  |
| Gender (n=905) |  |  |  |  |
| *Female* | 630 (77.5) | 67 (72.8) | 0.44 | - |
| *Male* | 179 (22.0) | 25 (27.2) |  |  |
| *Non-binary* | 4 (0.5) | 0 (0.0) |  |  |
| Time since first naturopathic qualification (n=901) |  |  |  |  |
| *Less than 5 years* | 254 (31.3) | 50 (55.6) | <0.001 | 0.1578 |
| *Between 5 and 10 years* | 164 (20.2) | 14 (15.6) |  |  |
| *Between 10 and 15 years* | 161 (19.9) | 8 (8.9) |  |  |
| *Between 15 and 20 years* | 100 (12.3) | 7 (7.8) |  |  |
| *More than 20 years* | 132 (16.3) | 11 (12.2) |  |  |
| World region where naturopathic training was completed (n=737) |  |  |  |  |
| *North American* | 293 (44.0) | 9 (12.7) | <0.001 | 0.3207 |
| *Latin American* | 74 (11.1) | 26 (36.6) |  |  |
| *South East Asian* | 28 (4.2) | 3 (4.2) |  |  |
| *European* | 130 (19.5) | 29 (40.9) |  |  |
| *Western Pacific* | 110 (16.5) | 3 (4.2) |  |  |
| *Other (African, Eastern Mediterranean, not specified)* | 31 (4.7) | 1 (1.4) |  |  |
| Currently in clinical practice | 674 (83.0) | 54 (62.1) | <0.001 | 0.1577 |
| Clinical environment (n=723) |  |  |  |  |
| *Solo clinic* | 261 (38.8) | 24 (47.1) | 0.283 | - |
| *Co-located with other health professionals but not other naturopaths* | 154 (22.9) | 10 (19.6) |  |  |
| *Co-located with other naturopaths but no other health professionals* | 52 (7.7) | 7 (13.7) |  |  |
| *Co-located with other naturopaths and other health professionals* | 149 (22.2) | 7 (13.7) |  |  |
| *Other clinical environment* | 56 (8.3) | 3 (5.9) |  |  |
| World region where clinical practice is located (n=591) |  |  |  |  |
| *North American* | 257 (46.5) | 4 (10.5) | <0.001 | 0.2886 |
| *Latin American* | 60 (10.9) | 17 (44.7) |  |  |
| *South East Asian* | 22 (4.0) | 1 (2.6) |  |  |
| *European* | 117 (21.2) | 14 (36.8) |  |  |
| *Western Pacific* | 71 (12.8) | 1 (2.6) |  |  |
| *Other (African, Eastern Mediterranean, not specified)* | 26 (4.7) | 1 (2.6) |  |  |

Cramer’s V: Effect size classified as negligible association (.00 and under .10); weak association (.10 and under .20); moderate association (.20 and under .40); relatively strong association (.40 and under .60); strong association (.60 and under .80) and very strong association (.80 and under 1.00), as reported by Rea and Parker (1992)(1)

1. Rea LM, Parker RA. Designing and conducting survey research: A comprehensive guide: John Wiley & Sons; 2014.
